# Transcranial magnetic stimulation for obsessive-compulsive disorder - preliminary results of an observational study

**DOI:** 10.1192/j.eurpsy.2024.1306

**Published:** 2024-08-27

**Authors:** I. Mangas Palma, J. Borges, E. Palha, H. Salgado, P. Alves, P. Sousa Martins, R. Moreira

**Affiliations:** Serviço de Psiquiatria, Centro Hospitalar Universitário de São João, Porto, Portugal

## Abstract

**Introduction:**

Obsessive-compulsive disorder (OCD) has a high prevalence and causes a significative reduction in functionality and quality of life.

First and second line treatment is ineffective in a variable percentage of patients. In such cases transcranial magnetic stimulation (TMS) may be considered.

**Objectives:**

The goal of this study is to evaluate the impact of TMS treatment on obsessive-compulsive, anxious and depressive symptomatology in patients with OCD.

**Methods:**

A prospective observational study was conducted, including all patients diagnosed with OCD who underwent TMS in the Psychiatry department of Centro Hospitalar Universitário de São João since March 2023.

Symptomatology was assessed using the Yale Brown Obsessive-Compulsive Scale (Y-BOCS), the Hamilton Anxiety Rating Scale (HAM-A) and the Hamilton Depression Rating Scale (HAM-D) before and after treatment.

Statistical analysis was performed using the SPSS-Statistics program. A significance level of 0.05 was considered.

**Results:**

As of October 31, 2023, nine individuals with OCD completed treatment with TMS, 33% male and with a median age of 40 years (range 33-57).

The median Y-BOCS score pre-TMS was 30 (range 20-33) and post-TMS 28 (range 16-34). The median difference was 2.5 (range -5-14) and was not statistically significant (p=0.128).

The median score on the HAM-A pre-TMS was 21 (range 9-41) and post-TMS 18 (range 11-24). The median difference was 0 points (range -4-21) and was not statistically significant (p=0.345).

The median HAM-D score pre-TMS was 26 (range 14-40) and post-TMS 19 (range 10-32). The median difference was 2.5 (range -3-20) and was not statistically significant (p=0.225).

**Conclusions:**

Preliminary findings suggest that the impact of TMS on obsessive-compulsive, anxious, and depressive symptomatology in patients with OCD does not appear to be clinically or statistically significant.

Further results are necessary to confirm this trend.

**Disclosure of Interest:**

None Declared

